# A hybrid coloboma and optic disc pit associated with macular retinoschisis

**DOI:** 10.1186/s12886-019-1221-z

**Published:** 2019-11-04

**Authors:** Ricardo Yuji Abe, Camila Ishii Iguma, Lorena Campos Wen

**Affiliations:** Hospital Oftalmológico de Brasília, SGAS 607 Avenida L2 Sul, Brasília, Distrito Federal Brazil

**Keywords:** Optic disc coloboma, Pit, Retinoschisis, Maculopathy, Optical coherence tomography

## Abstract

**Background:**

To report and describe an unusual case of a patient with optic disc pit in one eye and optic disc coloboma with a focal pit associated with macular retinoschisis in the other eye.

**Case presentation:**

A 21-year-old woman presented with optic disc pit in the right eye and optic disc coloboma with a focal pit like excavation in the left eye. Macular spectral domain optical coherence tomography (SD-OCT) of the left eye revealed macular retinoschisis, without serous detachment.

**Conclusions:**

Proper monitoring of patients with disc anomalies associated with maculopathy is mandatory. The use of OCT imaging during follow-up can help to identify involvement of the fovea or enlargement of the retinoschisis area.

## Background

Coloboma of the optic disc is a congenital ocular defect due to partial or complete failure of closure of the optic fissure during the sixth week of gestation and it can be associated with other congenital abnormalities [1]. Clinically, optic disc coloboma are seen as a bowl-shaped excavation located inferiorly with a normal superior neuroretinal rim with unilaterally and bilaterally occurrence with similar frequencies [2].

In 1882, Wiethe was the first author to describe congenital pit of the optic disc [3]. Even though pathophysiology of optic disc pits is not completely clear, some hypothesize that during ocular development, an abnormal differentiation of primitive epithelial papilla may lead to communication between the pit and subarachnoid space. The estimated prevalence of optic pits is between 0.02 and 0.19% [4, 5]. Some authors believe that optic disc pits and colobomas can share the same pathophysiology, while others authors considered optic disc pit as a variant of optic disc coloboma [6, 7]. Optic disc pits in general are less commonly associated with systemic associations and are not located near the optic fissure. Moreover, optic disc pits are usually unilateral and are sporadic in inheritance. In addition, optic disc pit are rarely found simultaneously with retinochoroidal or iris colobomas [5, 7].

It is well known that both optic disc coloboma and pit can develop macular complications [8]. The origin of the retinal fluid remains unclear. Possible sources include the vitreous cavity, the subarachnoid space and the orbital space surrounding the dura [7]. We present an unusual case of patient with optic disc pit in one eye and optic disc coloboma with a focal pit like excavation in the other eye associated with macular retinoschisis.

## Case presentation

A 21-year-old Caucasian woman was referred to our service as a glaucoma suspect due to the anomalous appearance of the optic nerve in both eyes. The patient had no visual complaints and no systemic pathologies were identified.

The patient denied any previous ocular pathology and surgeries. There was no family history of ocular diseases. On examination she had normal nasal bridge, logMAR best-corrected visual acuity was 0.0 in both eyes. Amsler grid was normal in both eyes. Anterior segment biomicroscopy, extrinsic ocular motility and pupillary light reflex were normal. Goldmann applanation tonometry was 12 mmHg in both eyes. Gonioscopic exam with Posner goniolens revealed open angles in both eyes.

**Fig. 1:**
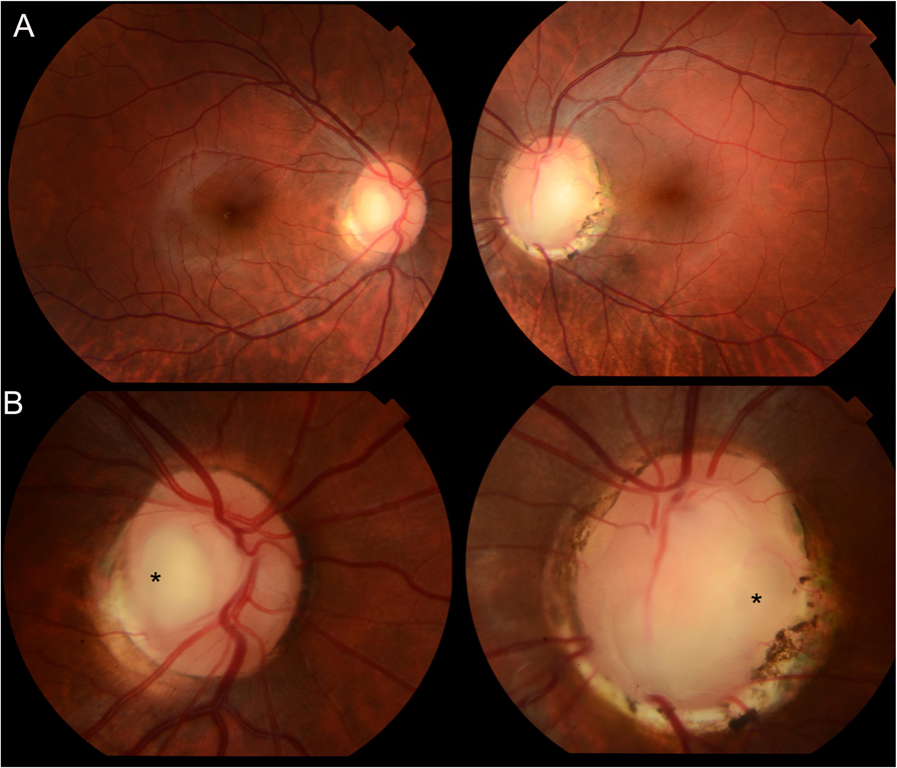
**a, b** Retinography (Topcon TRC-50DX, Japan) showing a localized depression in the temporal region of right eye (asterisk symbol) and a sharply delimited, glistening white, bowl-shaped excavation occupying an enlarged optic disc in the left eye. We also observe a focal pit-like excavation in the temporal quadrant of the disc (asterisk symbol) and macular folds on the internal limiting membrane in the left eye

**Fig. 2:**
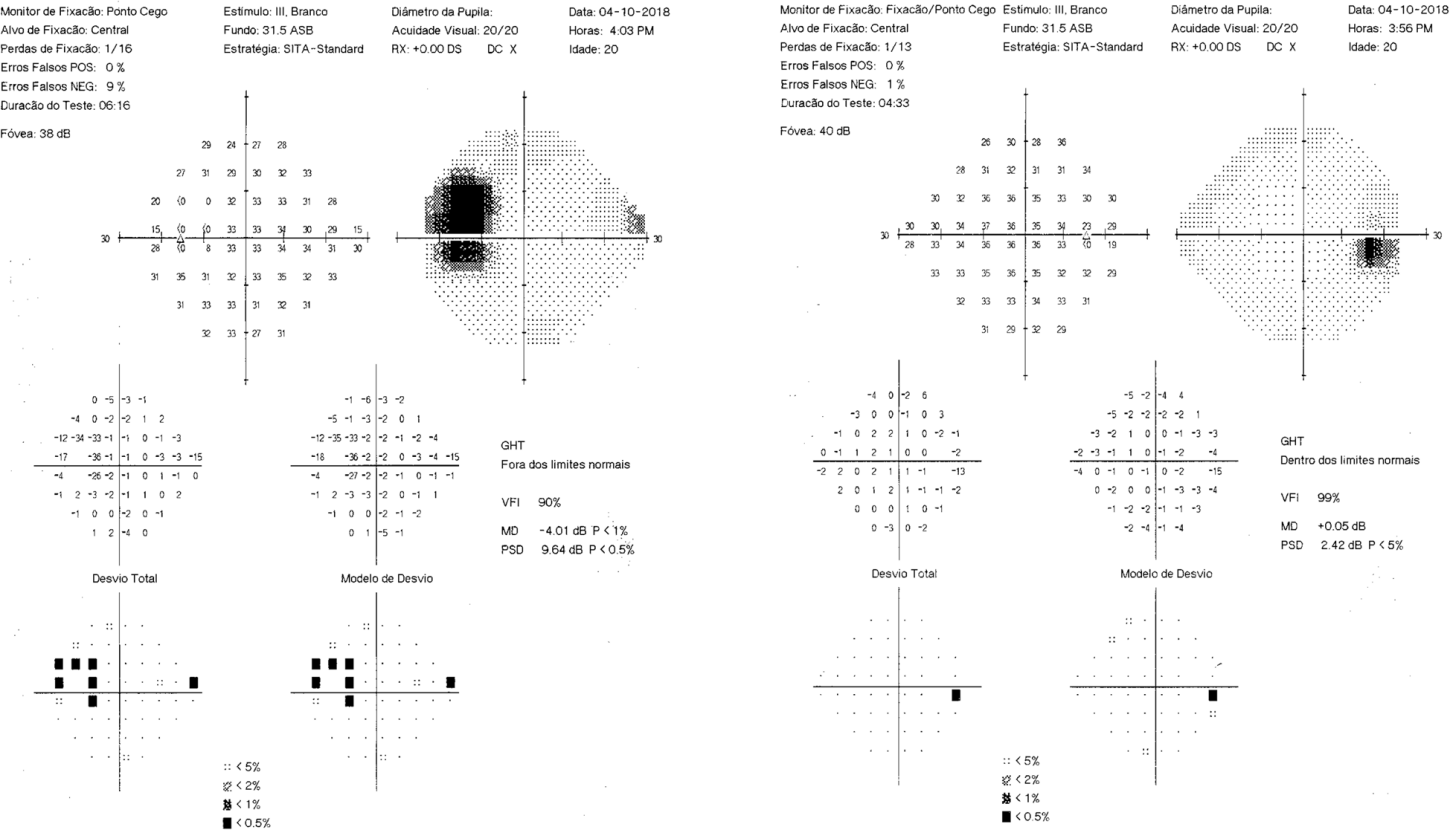
Visual fields (Humphrey Field Analyzer II, model 750i – Zeiss with 24–2 SITA Standard strategy). Enlargement of the blind spot on the left eye with no decreased retinal sensitivity in the central points of the visual field

Dilated fundus examination and retinography (Topcon TRC-50DX, Japan) revealed a localized depression in the temporal region of right and left eye (Fig. 1a and b, black asterisk). The left eye revealed a sharply delimited, glistening white, bowl-shaped excavation occupying an enlarged optic disc (Fig. 1a and b). The excavation was decentered inferiorly, with the inferior neuroretinal rim almost absent; while the superior neuroretinal rim was relatively spared. We also observe a focal pit-like excavations in the temporal quadrant of the disc and macular folds on the internal limiting membrane in the left eye (Fig. 1a and b). Visual fields were obtained using the Humphrey Field Analyzer II, model 750i (Zeiss) with 24–2 SITA Standard strategy. Exam of both eyes showed enlargement of the blind spot on the left eye with no decreased retinal sensitivity in the central points of the visual field (Fig. 2). Axial length obtained using ocular biometry (IOL Master 700, Zeiss) was 25,02 mm in the right eye and 24,15 mm in the left eye.

**Fig. 3:**
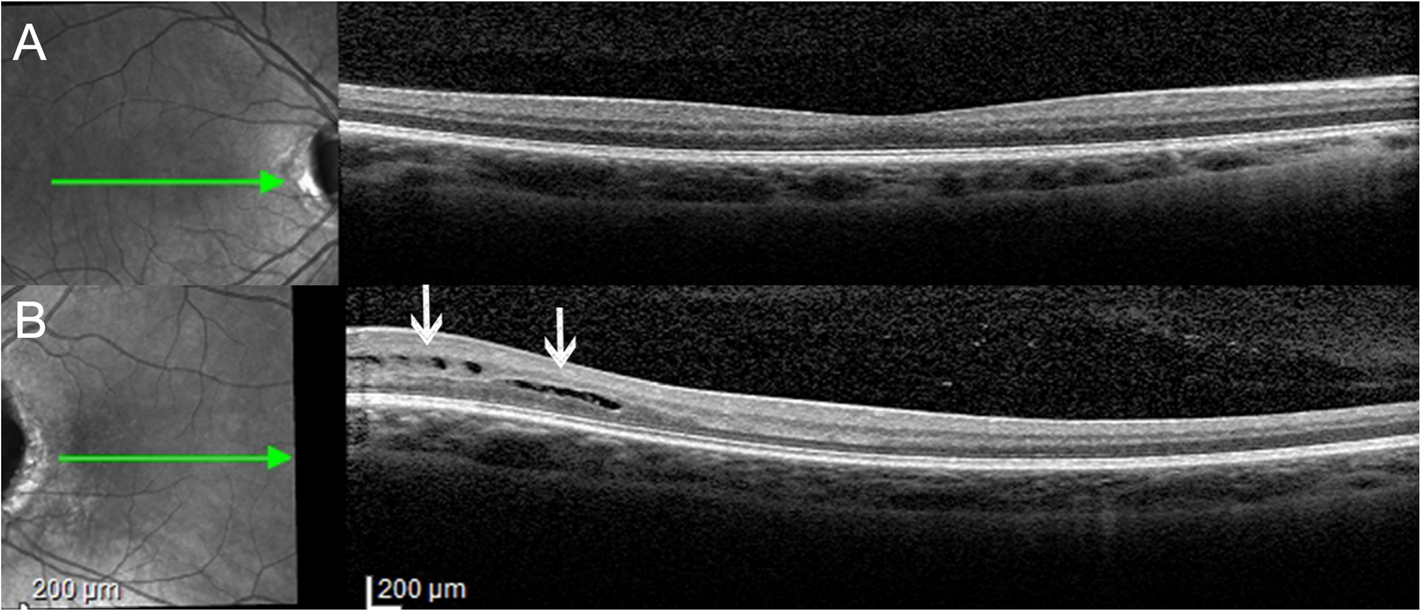
**a** Macular spectral domain optical coherence tomography (SD-OCT) (Spectralis; Heidelberg Engineering GmbH, Heidelberg, Germany) of the right eye with normal retinal thickness. **b** Macular SD-OCT of the left eye with intraretinal hyporreflective area, separating the neurosensory retina into two layers in the temporal perimacular region, suggesting retinoschisis (White arrows)

**Fig. 4:**
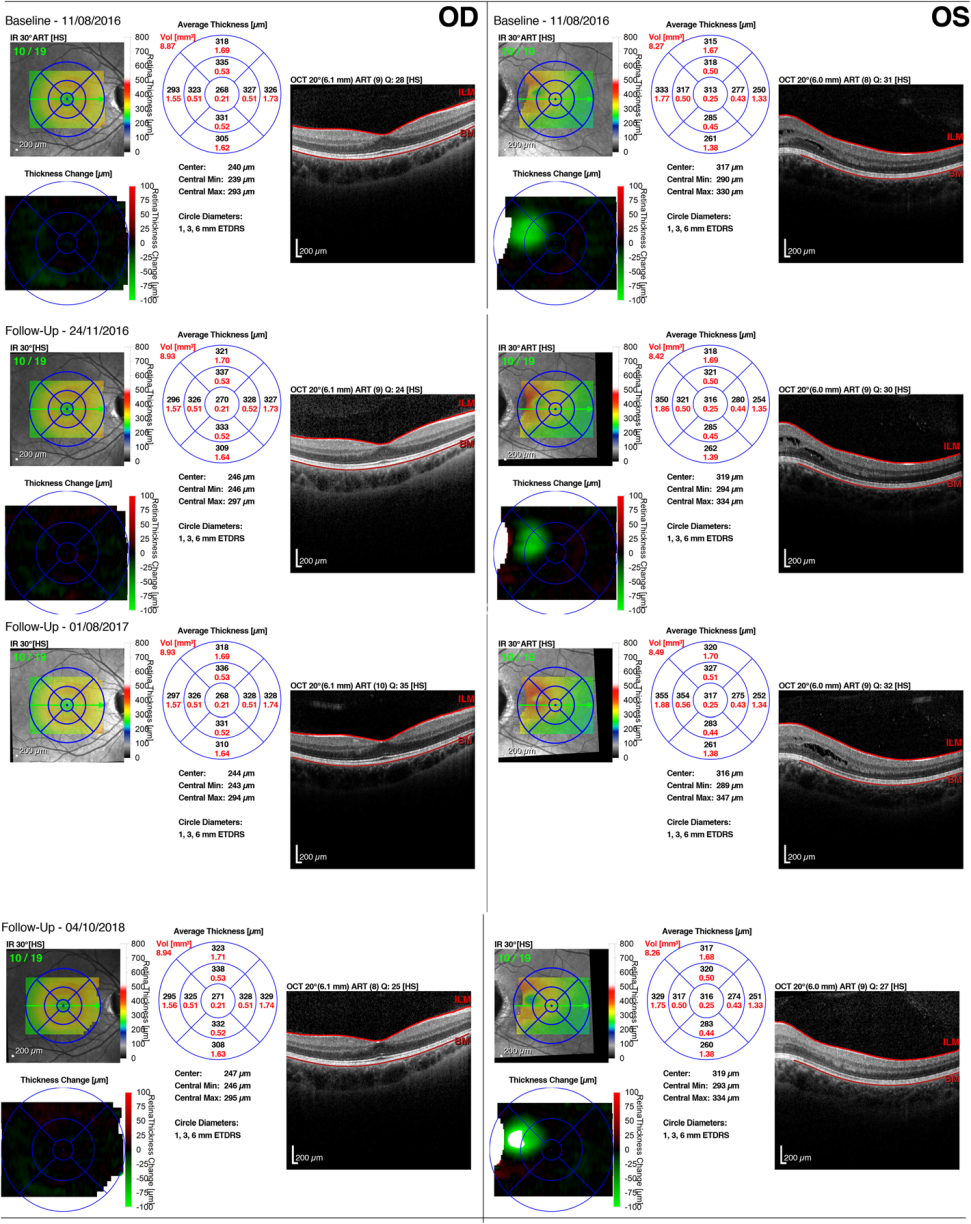
Macular spectral domain optical coherence tomography (SD-OCT) (Spectralis; Heidelberg Engineering GmbH, Heidelberg, Germany) with 2 year follow-up, without enlargement of the area of retinoschisis of the left eye and stability of the foveal thickness

Macular spectral-domain optical coherence tomography (SD-OCT) (Spectralis; Heidelberg Engineering GmbH, Heidelberg, Germany) showed no signs of retinoschisis (Fig. 3a) in the right eye. However, left eye showed intraretinal hyporreflective area, separating the neurosensory retina into two layers in the temporal perimacular region, suggesting a retinoschisis (Fig. 3b). After 2 year of follow-up, macular OCT showed no enlargement of the area of retinoschisis (Fig. 4) with central macular thickness remaining stable. The patient remained asymptomatic with unchanged visual acuity. Since the prevalence of cerebral abnormalities are relatively common in patients with optic disc coloboma, we performed magnetic resonance imaging but found no cerebral abnormalities [9].

We also examined the only sibling (brother) and her parents (father and mother) and none of them presented any abnormality at the ophthalmological examination.

## Discussion and conclusions

We describe a case of a patient with optic disc pit in the right eye and a coloboma with a pit-like excavation in the left eye. Congenital optic disc pits are seen alone or occasionally in combination with optic disc colobomas [10]. Previous case reports have described concomitant coloboma and optic disc pit in the same eye [11, 12]. In fact, incomplete development closure of the embryonic fissure might explain the coexistence of optic pits and colobomas as we found in the left eye of our patient. In addition to that, the patient presented isolated optic disc pit in the right eye.

Optic nerve colobomas can sometimes be misdiagnosed as morning glory discs [13]. In fact, the pathophysiology of an abnormal closure of the fetal fissure in morning glory discs can resemble similarities with optic disc coloboma [14]. However, in optic disc coloboma there is no central fibrous tuft and while in morning glory a variable amount of peripapillary pigment is evident, in optic disc coloboma minimal to none pigment is seen. In addition to that, morning glory optic discs are typically unilateral [15]. Congenital anomalies of the optic disc can mimic glaucoma due to overlapping clinical features. In fact, Takkar et al. described a case of an optic disc coloboma in association with optic disc pit in a 6-year-old child, who was being treated as developmental glaucoma with topical glaucoma medications [16].

According to Fig. 1, there is a small, craterlike depression within the temporal side of the colobomatous disc of the left eye. Using SD-OCT, we were able to identify a macular elevation with separation of the retinal layers. Savell et al. investigated fifteen members of a family with congenital colobomas [17]. Either macular or extra macular serous detachment or sequelae were present in 21 of the 30 affected eyes. Hotta et al. described a case of patient with choroidal and optic disc colobomas associated with an uncommon variant of macular detachment and retinoschisis [18]. According to the authors, the schisis-like separation found in optic disc colobomas and pits might be similar.

Although optic nerve head pits and colobomas are stationary, the associated retinal abnormalities may be progressive [8]. Our patient maintained good visual acuity in both eyes without any complaints, so we decided for continuous follow-up without further intervention. However evidence suggests that sensory macular retinal detachment can become manifest after the age of 20 [19]. Liconff described that the communication between the optic nerve pit and retina can cause a schisis-like separation of the inner retinal layers. In addition to that, a total separation of the outer retinal layers (full thickness macular detachment) appears to be secondary and associated with an outer lamellar macular hole [20]. Thus, careful observation and patient orientation is mandatory.

Some authors speculated that macular vitreous traction can be responsible for the detachment [21]. Thus, vitrectomy with or without peeling of the internal limiting membrane, laser photocoagulation, and gas tamponade can be used to minimize or eliminate vitreous traction [22]. A recent report found that vitrectomy alone without gas tamponade or endolaser was successful in treating the macular detachment [23].

Even though our patient maintains good visual acuity, we did not perform swept-source OCT to analyze vitreoretinal interface and identify an abnormal vitreous traction. Yokoi et al. using fundus photographs and swept-source OCT images evaluated seven eyes of six patients with maculopathy associated with optic disc pits [24]. The vitreous at the vitreoretinal interface was visualized by reconstructing three-dimensional swept-source-OCT images. Their findings suggested that an abnormal traction of the vitreous due to an abnormality of the might contribute to the maculopathy [24].

This case report described an unusual case of a patient with optic disc pit in the right eye and a spectrum of a hybrid anomaly (coloboma and pit-like exacavation associated) of the optic nerve in the left eye with macular retinoschisis. With proper follow up we highlight the importance of monitoring visual acuity and retinal changes with OCT.

## Data Availability

Not applicable.

## Abbreviation

SD-OCT: Macular spectral domain optical coherence tomography
